# Hydroxysafflor yellow A alleviates oxidative stress and inflammatory damage in the livers of mice with nonalcoholic fatty liver disease and modulates gut microbiota

**DOI:** 10.3389/fphar.2025.1568608

**Published:** 2025-06-06

**Authors:** Liang Wu, Xueyun Dong, Wen Sun, Tan Jiajun, Asmaa Ali, Jiayuan He, Pingping Wang

**Affiliations:** ^1^ Department of Laboratory Medicine, School of Medicine, Jiangsu University, Zhenjiang, China; ^2^ Department of Laboratory Medicine, Taizhou Second People’s Hospital, Taizhou, China; ^3^ Department of Critical Care Medicine, Jurong Hospital, Afliated to Jiangsu University, Zhenjiang, China; ^4^ Department of Pulmonary Medicine, Abbassia Chest Hospital, Cairo, Egypt; ^5^ Health Testing Center, Zhenjiang Center for Disease Control and Prevention, Zhenjiang, China

**Keywords:** hydroxysafflor yellow A, non-alcoholic fatty liver, oxidative stress, gut microbiota, amino acid anabolism

## Abstract

**Introduction:**

Hydroxysafflor yellow A (HSYA), its primary bioactive metabolite of *Carthamus tinctorius L*. (safflower), has shown therapeutic potential in various inflammatory diseases. However, its role in alleviating inflammation and oxidative stress in non-alcoholic fatty liver disease (NAFLD) remains unclear. This study investigates the therapeutic effects of HSYA in mice with NAFLD, focusing on its impact on gut microbiota and serum non-targeted metabolomics to elucidate the mechanisms underlying its efficacy.

**Methods:**

NAFLD was induced in mice using a high-fat diet (HFD), followed by intragastric administration of hydroxysafflor yellow A (HSYA). Serum levels of alanine aminotransferase (ALT), aspartate aminotransferase (AST), total cholesterol (TC), and triglycerides (TG) were quantified to evaluate liver function and lipid metabolism. Oxidative stress markers, including superoxide dismutase (SOD) activity and malondialdehyde (MDA) concentration, were also assessed. The pro-inflammatory cytokines IL-6, TNF-α, and IL-1β in serum were measured using ELISA. The hepatic expression of NLRP3 inflammasome and its downstream effector, Caspase-1, was analyzed by Western blot. Histopathological examination of liver tissues was performed using hematoxylin and eosin (H&E) staining to evaluate structural damage. Furthermore, alterations in the gut microbiota composition were characterized via 16S rDNA sequencing of fecal samples. Untargeted metabolomics was conducted to identify serum metabolic variations and elucidate enriched metabolic pathways associated with HSYA treatment.

**Results:**

HSYA significantly inhibited HFD-induced weight gain and alleviated liver inflammation. It reduced serum levels of alanine aminotransferase (ALT), aspartate aminotransferase (AST) and triglycerides (TG) (*P* < 0.05). HSYA administration decreased hepatic mRNA and protein expression of nucleotide binding oligomerization domain like receptor protein 3 (NLRP3), Caspase-1 and interleukin - 1β (IL-1β) while increasing superoxide dismutase (SOD) activity (*P* < 0.05). Gut microbiota analysis revealed a significant increase in the abundance of *Turicibacter*, while a reduction of *Ruminococcus*. Serum metabolomics identified a reduction in inflammation-associated metabolites, such as phenylalanine and tyrosine, alongside enhanced phenylalanine and tyrosine biosynthesis pathways.

**Discussion:**

HSYA demonstrates potent anti-inflammatory and antioxidant effects, effectively mitigating liver inflammation and oxidative stress in NAFLD mice. Its therapeutic mechanisms may involve modulating gut microbiota and regulating serum phenylalanine and tyrosine metabolism, offering insights into its potential as a treatment for NAFLD.

## 1 Introduction

Safflower (*Carthamus tinctorius* L.), a member of the Asteraceae family, is a traditional Chinese herbal medicine widely used for the treatment of cardiovascular and cerebrovascular diseases (Xue et al., 2021). Its therapeutic properties include scavenging oxygen free radicals and reducing inflammation (Wu et al., 2021). The primary bioactive compounds in safflower are its yellow pigments, including safflower yellow A, safflower yellow B and hydroxysafflower yellow A (HSYA), with HSYA accounting for approximately 85% of the total yellow pigments (Zhao et al., 2020; Wang et al., 2021). HSYA has been extensively investigated for its cardiovascular and cerebrovascular benefits, particularly its ability to eliminate oxygen-free radicals and suppress inflammatory cell infiltration (Mani et al., 2020).

Non-alcoholic fatty liver disease (NAFLD) is the most prevalent chronic liver disease globally (Paternostro and Trauner, 2022), characterised by excessive fat accumulation in more than 5% of liver cells in the absence of alcohol consumption, viral infections, or drug-induced liver damage (Bence and Birnbaum, 2021; Tilg et al., 2021). NAFLD affects approximately 25% of the global population, with obesity being a key risk factor (Riazi et al., 2022; Wong et al., 2023). It is strongly associated with insulin resistance, type 2 diabetes and metabolic syndrome, with a prevalence exceeding 50% among individuals with type 2 diabetes (Muzurović et al., 2021; Kosmalski et al., 2023). Excessive hepatic fat accumulation disrupts metabolic processes, leading to increased reactive oxygen species (ROS) production, mitochondrial endoplasmic reticulum stress and inflammatory damage (Lim et al., 2021; Čolak and Pap, 2021). Patients with NAFLD often exhibit impaired antioxidant defences, characterised by reduced blood levels of antioxidants (e.g., vitamins E and C) and elevated lipid peroxidation products and systemic oxidative stress (Arroyave-Ospina et al., 2021; Yasmin et al., 2021).

Natural dietary antioxidants have shown promise in mitigating NAFLD-related oxidative stress, mitochondrial dysfunction, insulin resistance and inflammation (Sohouli et al., 2020; Mohammadian et al., 2024). While HSYA is recognised as a potent antioxidant, its therapeutic potential for NAFLD remains underexplored. This study aims to investigate the effects and mechanisms of HSYA using a high-fat diet-induced NAFLD mouse model, offering new insights into its potential as a treatment for NAFLD.

## 2 Materials and methods

### 2.1 Animals, NAFLD model and treatment

Following the methods reported by Sun et al. (2023), an NAFLD model was established in ICR mice using a high-fat diet (HFD). Detailed experimental procedures are provided in the Supplementary Material S1. Male ICR mice (6 weeks old; 25 g ± 4 g) were obtained from Jiangsu Wukong Biotechnology Co., Ltd. (Nanjing, China) and housed at the Jiangsu University Experimental Animal Centre (Ethics Committee of Jiangsu University approval number: UJS-IACUC-AP-2022032011). Forty mice were randomly assigned to four groups (10 mice per group): the normal control group (NC), the NAFLD group, the low-dose HSYA group (HSYAL) and the high-dose HSYA group (HSYAH). The NC group was fed a standard diet, while the other three groups were fed HFD, with 40% of energy derived from fat. HSYA (purity >98%) was provided by Shanghai Shifeng Biological Co., Ltd. (Shanghai, China). Based on previous studies (Liu et al., 2018; Lee et al., 2020), HSYAL and HSYAH groups received oral doses of 60 mg/kg and 120 mg/kg HSYA, respectively, while the NC and NAFLD groups were administered an equal volume of saline daily. The experiment was concluded at the end of the 12th week, and samples were collected for analysis.

### 2.2 Serum biochemical analysis, qPCR, Western blotting and ELISA assay

Based on Sun et al.'s method (Sun et al., 2023), serum biochemical indicators, including triglycerides (TG), total cholesterol (TC), alanine aminotransferase (ALT), aspartate aminotransferase (AST), superoxide dismutase (SOD) and malondialdehyde (MDA), were measured. Assay kits were purchased from Nanjing Jiancheng Bioengineering Institute (Nanjing, China). Detailed procedures are available in the Supplementary Material S2. The expressions of *IL-1*β, *NLRP3* and *Caspase-1* in liver tissues were assessed using quantitative PCR (qPCR) and Western blotting, as described by Sun et al. Primer sequences and qPCR protocols are provided in the Supplementary Material S3. Serum concentrations of IL-6, TNF-α and IL-1β were quantified using ELISA kits (Meimian, Yancheng, China), strictly adhering to the manufacturer’s instructions.

### 2.3 Hematoxylin-eosin (HE) staining

Liver tissue samples (∼2 mm^3^) were fixed in 4% paraformaldehyde for 16 h. Fixed samples were then sent to Shanghai Labway Medical Testing Co., Ltd. (Shanghai, China) for HE staining. Tissue sections were prepared at a thickness of 4 μm. Stained sections were examined under a microscope to evaluate liver inflammation in each group.

### 2.4 16S rRNA gene sequencing

Colon contents from the mice were sent to Ekemo Tech Group Co., Ltd. (Shenzhen, China) for bacterial 16S rRNA gene sequencing. The resulting gene sequence data were annotated by species and analysed using bioinformatics methods. Principal component analysis (PCA) and orthogonal partial least squares discriminant analysis (OPLS-DA) were performed to evaluate the model’s effectiveness and predictability. Cluster heatmap analysis revealed that HSYA significantly influenced the gut microbiota composition in mice.

### 2.5 Non-targeted metabolomic analysis

Mouse serum samples were analysed for untargeted metabolomics by Ekemo Tech Group Co., Ltd. (Shenzhen, China) using the UPLC-Q-TOF-MS/MS platform (Agilent Technologies, Waldbronn, Germany). Multivariate statistical analyses, including PCA and OPLS-DA, were conducted to process the metabolomics data. Variable importance in projection (VIP) values for each metabolite were calculated using the MetaX software. Differentially expressed metabolites were identified based on the criteria VIP >1.0 and *P* < 0.05. These metabolites were then subjected to the Kyoto Encyclopedia of Genes and Genomes (KEGG) pathway enrichment analysis and associated network analysis.

### 2.6 Statistical analysis

Statistical analyses were performed using SPSS 20.0 (SPSS, Chicago, IL, USA), and results are expressed as mean ± standard deviation (SD). Group comparisons were conducted using *t*-tests and one-way ANOVA to assess statistical significance. A *P*-value <0.05 was considered statistically significant. Data visualisation and further analyses were carried out using GraphPad Prism software and the Bioincloud online platform (https://bioincloud.tech/).

## 3 Results

### 3.1 Weight and liver changes in NAFLD mice

**FIGURE 1:**
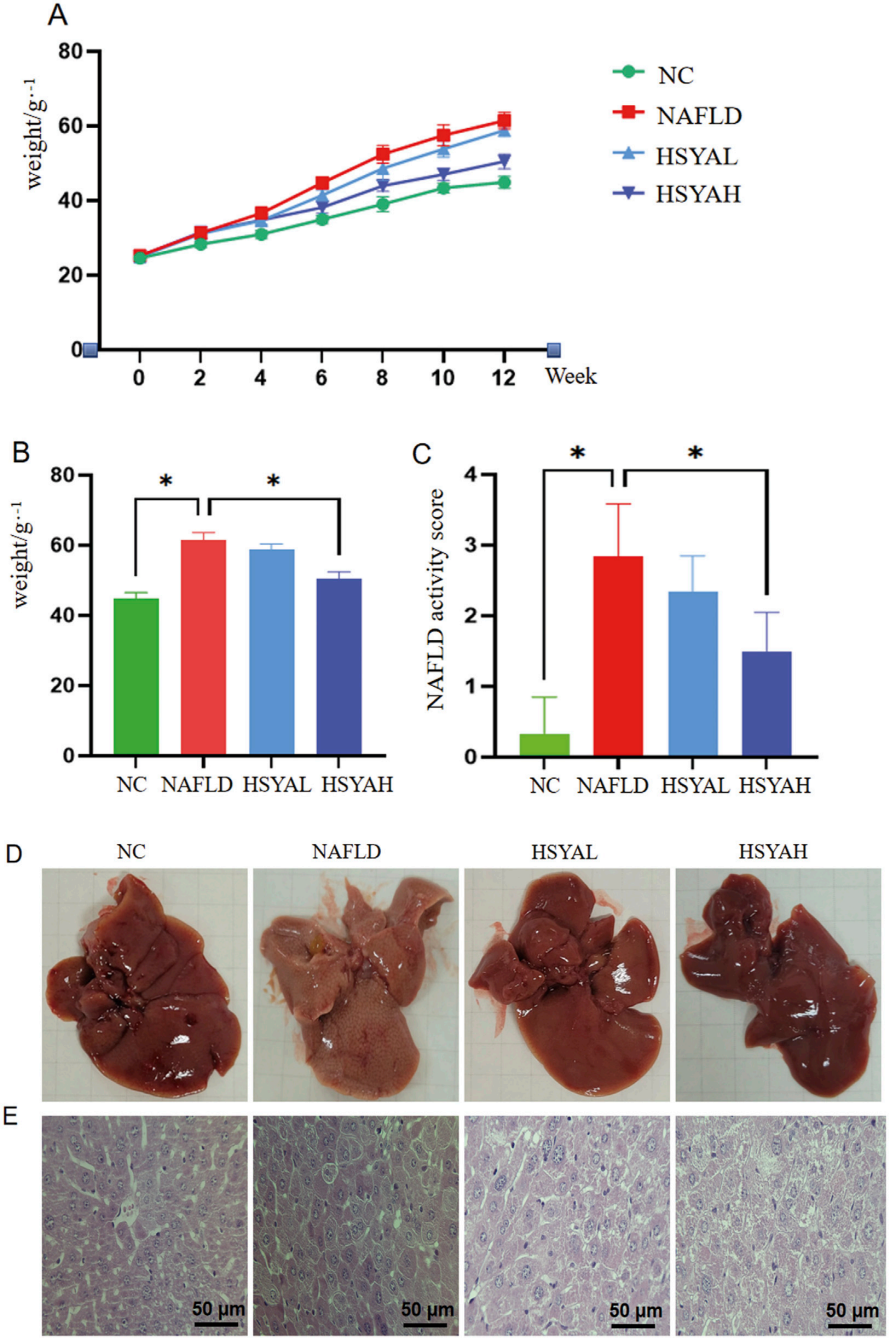
Changes in body weight and liver damage in mice. ICR mice were induced with NAFLD by high-fat diet and treated with high and low concentrations of HYSA (60 mg/kg and 120 mg/kg). Changes in body weight **(A,B)** and NAFLD activity score **(C)** of each group of mice were monitored during the experiment, and the color **(D)** and structural changes **(E)** of the liver in each group of mice were observed at the end of the experiment (n = 10).

Both low and high doses of HSYA demonstrated significant therapeutic effects on body weight and liver pathology in mice with NAFLD. The HFD led to marked weight gain and the development of fatty liver disease in the mice. By week 8, the body weight of the HSYAH group was significantly lower than that of the NAFLD group (*P* < 0.05), while no significant change was observed in the HSYAL group (*P* < 0.05) (Figures 1A,B).

Mice in the NAFLD group exhibited pale, greasy livers, whereas both low and high doses of HSYA significantly improved liver damage, with the HSYAH group showing more pronounced improvement (Figures 1C,D). Staining revealed that liver cells in the NAFLD group exhibited prominent lipid droplets, inflammatory cell infiltration and cell necrosis. Following HSYA intervention, liver pathology in both the HSYAH and HSYAL groups showed significant recovery, with a marked reduction in necrotic cells (Figure 1E).

### 3.2 HSYA improves oxidative stress and blood lipid levels in NAFLD mice

**FIGURE 2:**
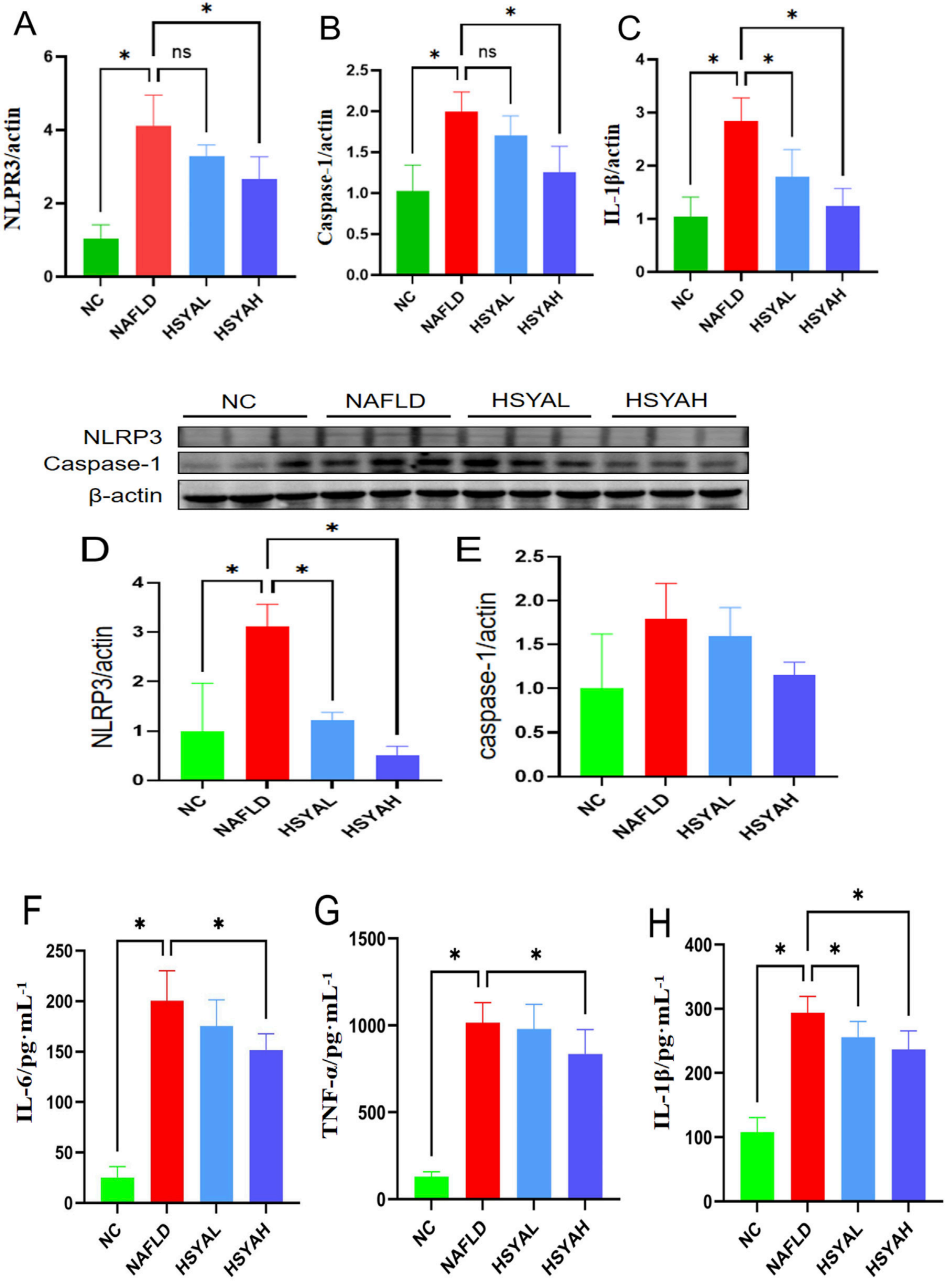
The inflammatory factors in mice were detected using qPCR **(A–C)**, Western blotting **(D,E)**, and ELISA methods **(F–H)**. The qPCR method was used to detect the mRNA expression levels of NLRP3, Caspase-1, and IL-1β in the liver (n = 6). The Western blotting method was used to detect the expression levels of NLRP3 and Caspase-1 in the liver (n = 3). The ELISA method was used to detect the expression levels of IL-6, TNF-α, and IL-1β in the serum (n = 3). *: *P* < 0.05; ns: *P* > 0.05.

**FIGURE 3:**
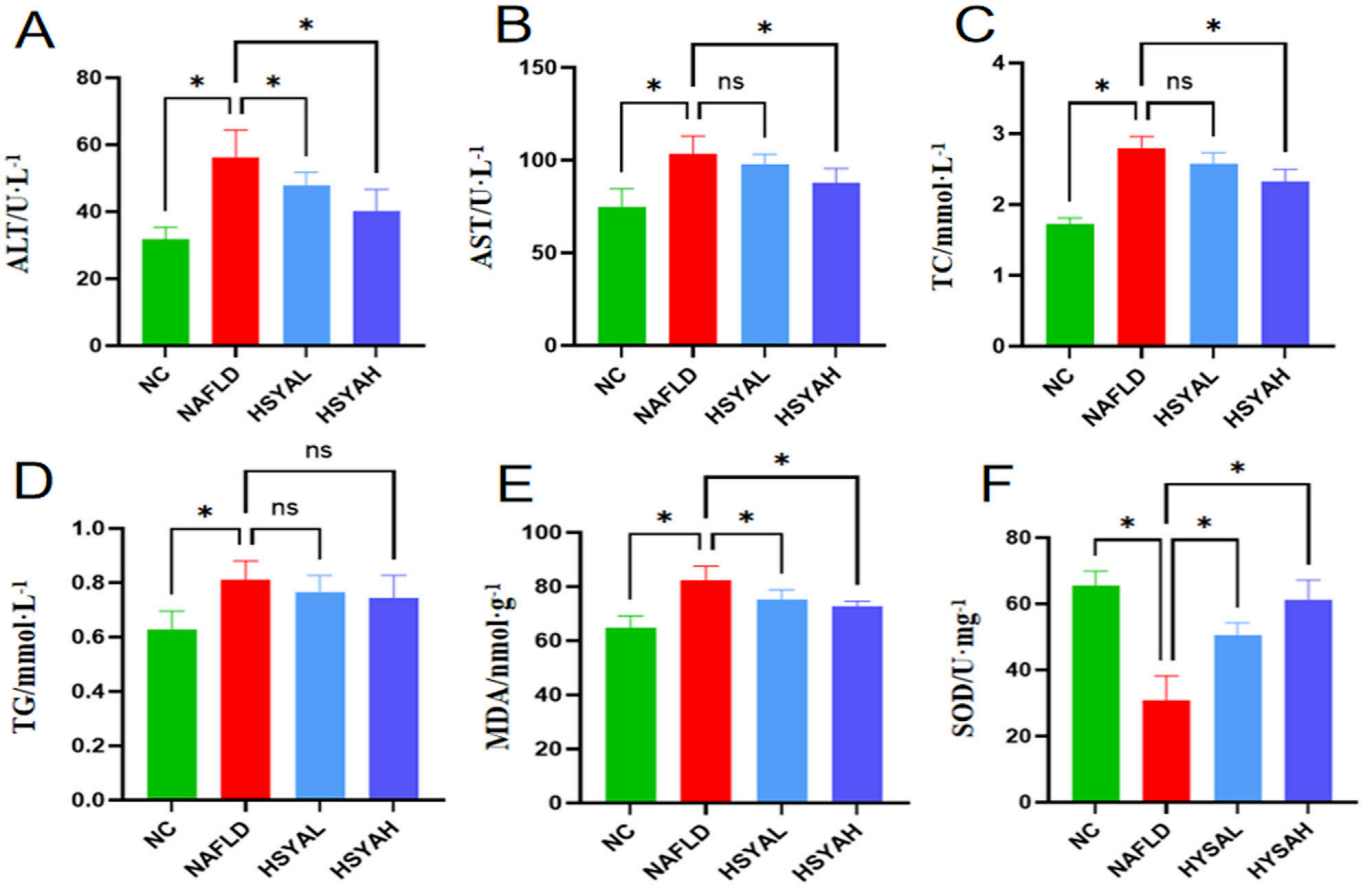
Liver function indicators ALT and AST levels in serum **(A,B)**, n = 6. The blood lipid related indicators TC and TG levels **(C,D)**, n = 6. The oxidative stress response indicators MDA and SOD levels **(E,F)**, n = 6, and. *: *P* < 0.05; ns: *P* > 0.05.

HFD significantly elevated the expressions of NLRP3, Caspase-1 and IL-1β in the liver (*P* < 0.05) (Figure 2). Serum levels of IL-6, IL-1β and TNF-α were also significantly increased (*P* < 0 0.05) (Figure 2), while serum SOD levels were significantly reduced and MDA levels were significantly elevated, indicating increased oxidative stress (*P* < 0.05) (Figure 3). Moreover, serum TG, TC, ALT and AST levels were significantly elevated (*P* < 0.05), indicating that the HFD induced lipid metabolism disorders, leading to fat deposition in the liver and the onset of fatty liver disease (Figure 3).

Following HSYA intervention, the mRNA expression levels of NLRP3, Caspase-1 and IL-1β in the liver were significantly reduced (*P* < 0.05), and protein expression levels of NLRP3 and Caspase-1 were also significantly decreased (*P* < 0.05). Serum levels of IL-6, IL-1β and TNF-α were also significantly decreased (*P* < 0.05) (Figure 2), indicating that high-dose HSYA significantly inhibits the inflammatory response in the liver. Furthermore, oxidative stress markers SOD and MDA showed significant improvement, indicating that both low and high doses of HSYA effectively reduced oxidative stress induced by the HFD (*P* < 0.05) (Figure 3). After HSYA intervention, there was no significant change in TG levels, but high-dose HSYA significantly reduced TC levels, and ALT and AST levels showed significant improvement (*P* < 0.05), suggesting that high-dose HSYA effectively alleviated liver damage and oxidative stress in NAFLD mice (Figure 3).

### 3.3 HSYA reshapes intestinal microbiota and serum metabolomics in NAFLD mice

Given that high-dose HSYA (120 mg/kg) effectively suppresses oxidative stress and exhibits a pronounced hepatoprotective effect in NAFLD mice, we further investigated its impact on gut microbiota and serum metabolomics.

**FIGURE 4:**
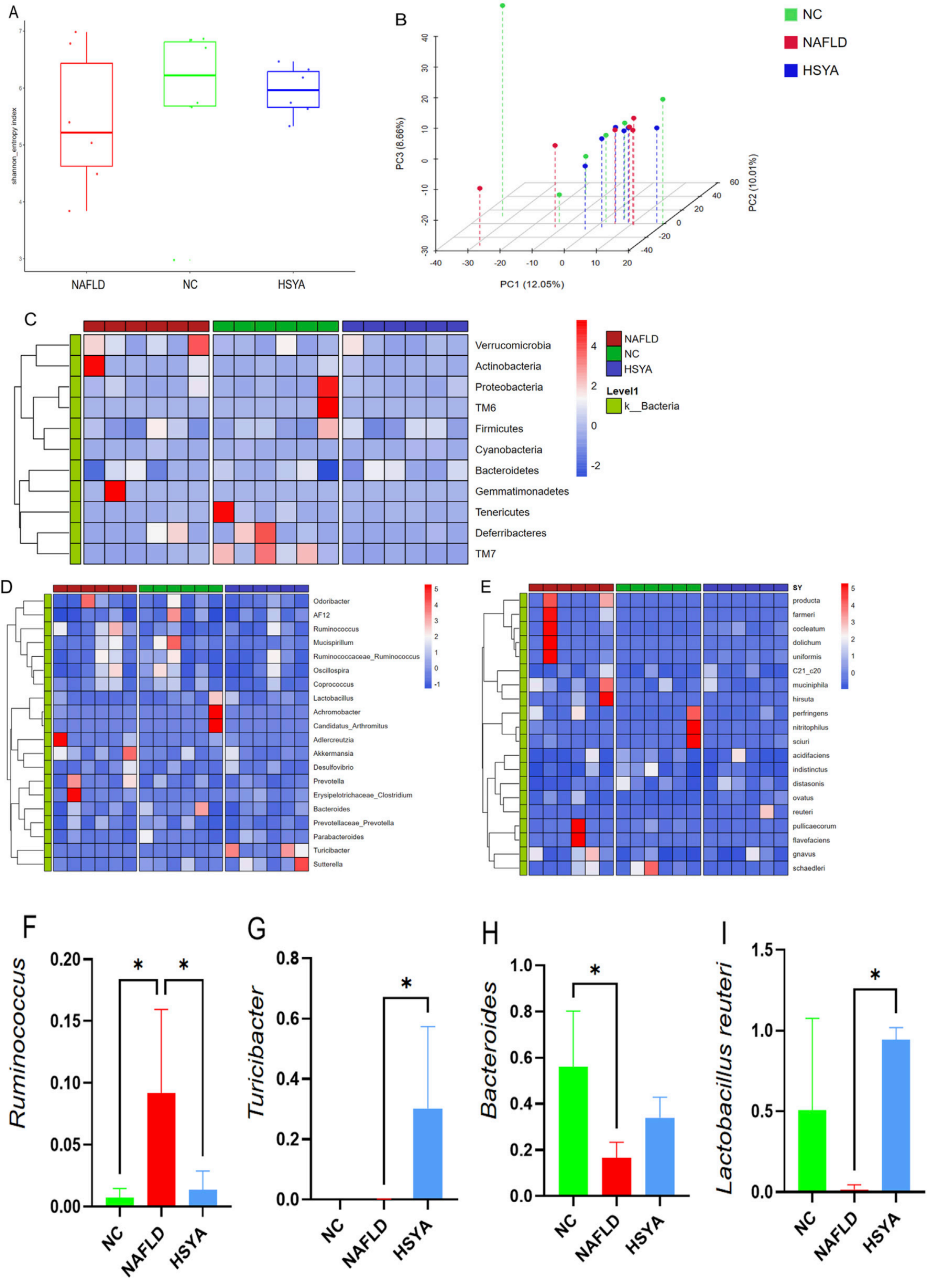
Using 16S rDNA technology to detect the gut microbiota of mice (n = 6). The α diversity **(A)** and β diversity **(B)** of gut microbiota in each group of mice. Further cluster heatmap analysis revealed significant changes in the abundance of bacteria at the phylum **(C)**, genus **(D)**, and species **(E)** levels in the gut microbiota of mice. Intestinal microbes with significant differences in abundance **(F–I)**. *: *P* < 0.05.

The results indicate that HSYA does not significantly influence the α-diversity of the intestinal microbiota in NAFLD mice, as measured by the Shannon index (*P* > 0.05). However, PCA revealed distinct separation among the three sample groups (Figures 4A,B).

Analyze the composition of mouse gut microbiota at the phylum, genus, and species levels using cluster heatmap method (Figures 4C–E). At the genus level, NAFLD mice demonstrated increased relative abundances of *Ruminococcus*, with concurrent reductions in *Bacteroides*; HSYA treatment significantly increased the abundance of *Turicibacter* levels, with concurrent reduction in *Ruminococcus* (*P* < 0.05). At the species level, HSYA treatment significantly increased the abundance of *Lactobacillus reuteri* (*P* < 0.05) (Figures 4F-I).

**FIGURE 5:**
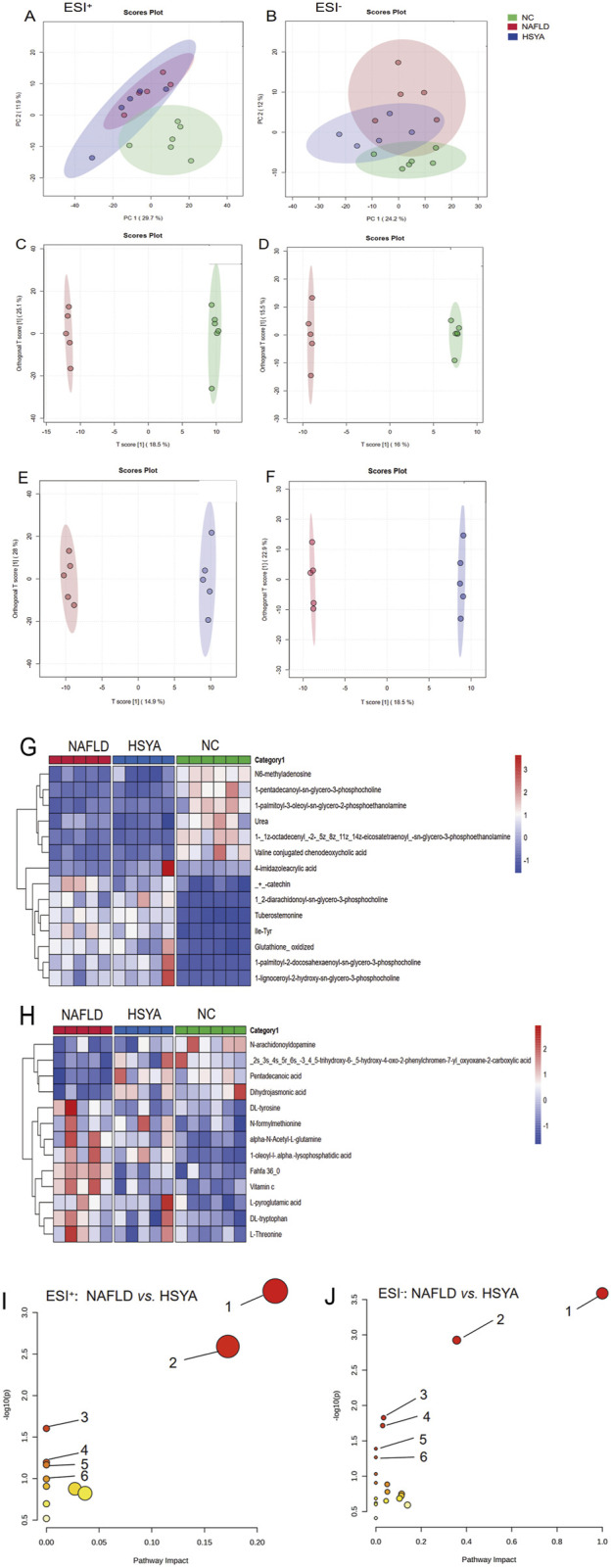
Using non-targeted metabolomics technology to detect the serum metabolomics of mice (n = 6). In ESI^+^ mode, the mouse serum PCA plot shows that samples from each group were clearly clustered **(A)**; in ESI^−^ mode, samples from each group were clustered and separated **(B)**. Further analysis using OPLS-DA **(C-F)** identified different metabolites in the ESI^+^ mode **(G)** and the ESI^−^ mode **(H)** based on VIP>1 and *P* < 0.05 criteria. Enrichment of the above different metabolites obtained the metabolic pathways related to NAFLD in mice regulated by HSYA. ESI^+^ **(I)**: ① biosynthesis of phenylalanine, tyrosine, and tryptophan; ② phenylalanine metabolism; ③ niacin and niacinamide metabolism; ④ starch and sucrose metabolism; ⑤ glycine, serine, and threonine metabolism; ⑥ tryptophan metabolism. ESI^−^ **(J)**: ① biosynthesis of phenylalanine, tyrosine, and tryptophan; ② pyrimidine metabolism; ③ phenylalanine metabolism; ④ ascorbic acid and aldaric acid metabolism; ⑤ biotin metabolism; ⑥ histidine metabolism.

Metabolomic analyses using electrospray ionisation in both positive (ESI+) and negative (ESI−) modes revealed distinct clustering and separation among the NC, NAFLD and HSYA groups. This confirmed the successful establishment of the NAFLD model. In the ESI− mode, the HSYA group exhibited closer alignment with the NC group, highlighting HSYA’s significant regulatory effects on metabolic disturbances in NAFLD mice (Figures 5A,B). OPLS-DA identified numerous differential metabolites based on VIP > 1 and *P* < 0.05 criteria (Figures 5C–H; Tables 1,2). Pathway enrichment analysis revealed that HSYA modulated several metabolic pathways, including phenylalanine, tyrosine and tryptophan biosynthesis, starch and sucrose metabolism and phenylalanine metabolism (Figures 5I,J).

**TABLE 1:** Differential metabolites in mouse serum in the ESI^+^ mode (NAFLD group vs. HSYA group).

NO	Metabolite	Formula	HMDB	m/z	rt (s)	Which-max
1	L-Anserine	C10H16N4O3	HMDB0000194	282.15447	431.497	↑
2	Urea	CH4N2O	HMDB0000294	61.03924	93.3845	↓
3	4-imidazoleacrylic acid	C6H6N2O2	HMDB0000301	139.05004	304.984	↓
4	1-Stearoyl-2-oleoyl-sn-glycerol 3-phosphocholine (SOPC)	C44H86NO8P	HMDB0000564	832.58196	121.96	↑
5	Deoxycholic acid	C24H40O4	HMDB0000626	785.61096	42.913	↑
6	Trimethylamine n-oxide	C3H9NO	HMDB0000925	76.07505	338.9335	↓
7	DL-2-Aminooctanoic acid	C8H17NO2	HMDB0000991	201.15814	213.152	↓
8	Folinic acid	C20H23N7O7	HMDB0001562	474.17015	432.069	↑
9	(+)-catechin	C15H14O6	HMDB0002780	291.10577	418.518	↑
11	Glutathione, oxidized	C20H32N6O12S2	HMDB0003337	613.15574	515.461	↑
12	N6-methyladenosine	C11H15N5O4	HMDB0004044	282.11625	400.027	↓
14	1-(1z-octadecenyl)-2-(5z,8z,11z,14z-eicosatetraenoyl)-sn-glycero-3-phosphoethanolamine	C43H78NO7P	HMDB0005779	734.53258	39.3875	↓
15	Linoleoylcarnitine	C25H45NO4	HMDB0006469	424.33891	43.565	↓
16	Sialyl lewis x	C31H52N2O23	HMDB0006565	430.14241	308.297	↓
17	1-palmitoyl-2-docosahexaenoyl-sn-glycero-3-phosphocholine	C46H80NO8P	HMDB0007991	806.56728	40.5845	↑
18	1,2-diarachidonoyl-sn-glycero-3-phosphocholine	C48H81NO8P+	HMDB0008443	830.56344	122.992	↑
20	1-lignoceroyl-2-hydroxy-sn-glycero-3-phosphocholine	C32H66NO7P	HMDB0010405	608.45903	184.41	↑

**TABLE 2:** Differential metabolites in mouse serum in the ESI^−^ mode (NAFLD group vs. HSYA group).

NO	Metabolite	Formula	HMDB	m/z	rt(s)	Which-max
1	Vitamin c	C6H8O6	HMDB0000044	351.05442	431.348	↑
2	cis-Aconitate	C6H6O6	HMDB0000072	173.00803	467.6255	↑
3	DL-tyrosine	C9H11NO3	HMDB0000158	180.06549	333.17	↑
4	Phenylalanine	C9H11NO2	HMDB0000159	164.07081	298.4045	↑
5	L-Threonine	C4H9NO3	HMDB0000167	118.05035	403.657	↑
6	Indoleacetic acid	C10H9NO2	HMDB0000197	130.05026	397.1245	↑
7	L-pyroglutamic acid	C5H7NO3	HMDB0000267	128.03588	311.1905	↑
8	3-hydroxy-3-methylglutaric acid	C6H10O5	HMDB0000355	161.04463	403.563	↑
9	2-aminoadipic acid	C6H11NO4	HMDB0000510	160.06063	436.143	↑
10	Glutamine	C5H10N2O3	HMDB0000641	145.06161	424.263	↑
11	L-methionine	C5H11NO2S	HMDB0000696	148.04287	319.019	↑
12	Pentadecanoic acid	C15H30O2	HMDB0000826	241.21581	54.627	↑
13	N-formylmethionine	C6H11NO3S	HMDB0001015	176.03728	207.8135	↑
15	5-methoxytryptophan	C12H14N2O3	HMDB0002339	467.17127	35.727	↓
16	N-acetylserine	C5H9NO4	HMDB0002931	146.04511	312.919	↑
17	Glutathione disulfide	C20H32N6O12S2	HMDB0003337	611.1419	538.491	↓
18	2-oleoyl-1-stearoyl-sn-glycero-3-phosphoserine	C42H80NO10P	HMDB0010163	788.52802	41.042	↓
19	N-palmitoyl-d-erythro-dihydroceramide-1-phosphate	C34H70NO6P	HMDB0010698	618.47279	51.437	↓
20	1-palmitoyl-2-hydroxy-sn-glycero-3-phosphoethanolamine	C21H44NO7P	HMDB0011503	452.27493	185.345	↓
23	2-oleoyl-1-palmitoyl-sn-glycero-3-phosphoserine	C40H76NO10P	HMDB0012357	760.51583	65.53	↓
24	1,2-distearoyl-sn-glycero-3-phospho-l-serine	C42H82NO10P	HMDB0012378	773.52958	94.835	↓
26	DL-tryptophan	C11H12N2O2	HMDB0030396	203.08143	295.0945	↑
28	D-Mannose	C6H12O6	HMDB0062473	179.05691	313.3165	↑

### 3.4 Analysis of the correlation between gut microbiota and serum differential metabolites

**FIGURE 6:**
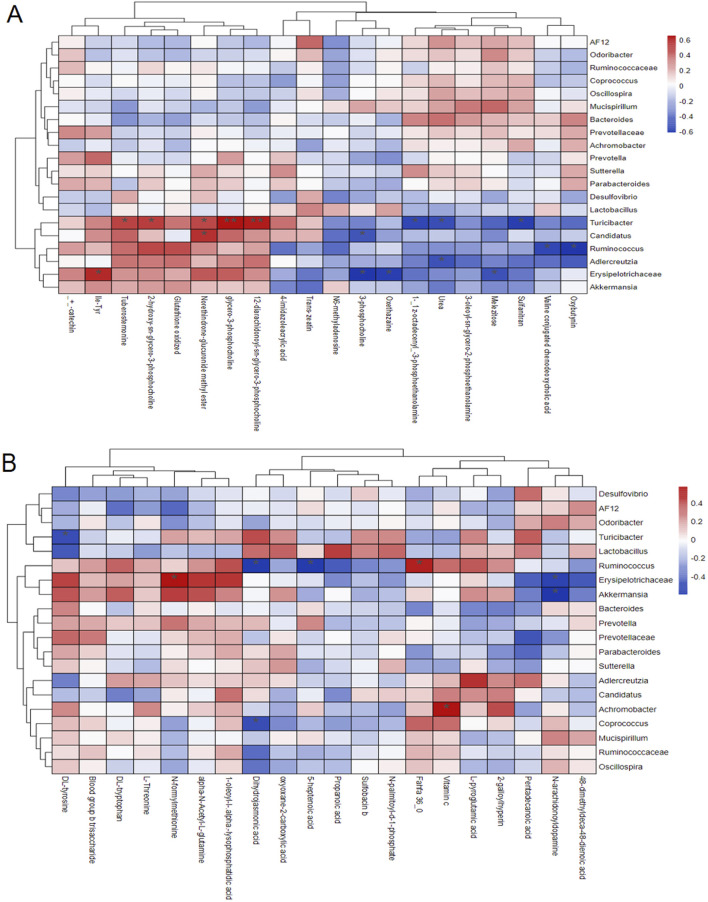
Pearson correlation analysis results of differences in gut microbiota and serum metabolites in mice under ESI^+^ mode **(A)** and ESI^−^ mode **(B)**.

Pearson correlation analysis revealed significant associations between the relative abundance of various gut microbiota and serum metabolites (Figure 6). The relative abundance of *Turicibacter* showed a positive correlation with phosphatidylcholine levels in mouse serum and a negative correlation with DL-tyrosine, sulfanilamide and urea levels. Similarly, the abundance of *Verrucomicrobia* was positively correlated with Fahfa:36.0 levels and negatively correlated with leucine and deoxycholic acid levels. Additionally, *Erysipelotrichaceae-Clostridium* abundance exhibited a positive correlation with N-formylmethionine levels and a negative correlation with trehalose and phosphatidylcholine levels.

## 4 Discussion

The pathogenesis of NAFLD is multifaceted, involving oxidative stress, lipid metabolism dysregulation and gut microbiota imbalance (Tilg et al., 2021). Although the progression of simple NAFLD is reversible, untreated chronic inflammation and oxidative stress in the liver can escalate the condition into non-alcoholic steatohepatitis (NASH) and eventually liver fibrosis, significantly worsening patient prognosis (Ramai et al., 2021; Tacke and Weiskirchen, 2021; Li J. et al., 2022).

HSYA, the primary bioactive metabolite of safflower (*C. tinctorius*), belongs to the flavonoid class of compounds (Zhao et al., 2020). Modern pharmacological research has demonstrated that HSYA exhibits anti-inflammatory, antioxidant, anti-apoptotic and protective effects on the cardiovascular and nervous systems (Kong et al., 2022; Yu et al., 2022; Wang et al., 2024). Animal studies have shown that HSYA enhances the expression of antioxidant enzymes, such as superoxide dismutase, in the liver (Lee et al., 2020; Yan et al., 2020; Yao et al., 2025). Furthermore, research using H_2_O_2_-induced HepG2 cells and oxidative stress models in adipocytes has indicated that HSYA promotes the expression of cellular antioxidant factors and enzymes, improving cellular resilience against oxidative stress (Yan et al., 2020). Our findings corroborate these results, demonstrating that oral administration of HSYA significantly reduces inflammatory responses and oxidative stress in high-fat diet-induced NAFLD mice. Notably, HSYA significantly decreased serum TC levels in these mice, although it had no significant effect on triglyceride (TG) levels. This discrepancy may be attributed to HSYA altering cholesterol distribution within the body. However, the current study utilised HE staining to evaluate liver pathology, which did not allow for a detailed analysis of lipid content. Future studies will employ Oil Red O staining to assess liver lipid profiles and investigate HSYA’s specific effects on cholesterol and other lipid distributions.

To further explore the mechanisms by which HSYA mitigates inflammation and liver damage in NAFLD, we analysed gut microbiota and serum non-targeted metabolomics in the experimental mice. HFD significantly increased the abundance of *Ruminococcus* in the intestines, consistent with previous findings of elevated *Ruminococcus* levels in the gut of patients with NAFLD (Lee et al., 2020; Pettinelli et al., 2022). HSYA treatment significantly reduced *Ruminococcus* abundance in the intestines of high-fat diet-fed mice while significantly increasing the abundance of beneficial *Turicibacter*.

Existing research indicates that both *Ruminococcus* and *Turicibacter* in the gut are involved in tryptophan metabolism. The abundance of *Ruminococcus* is positively correlated with the production of indoleamine 2,3-dioxygenase (IDO1) in the gut (Li et al., 2022b). It can activate macrophage IDO1 through the metabolite succinate, thereby activating macrophages and consuming tryptophan to produce a large amount of the pro-inflammatory metabolite kynurenine (Riazati et al., 2022; Ma et al., 2024). *Turicibacter* carries a homologous gene for tryptophan hydroxylase, which can catalyze the conversion of tryptophan into 5-hydroxytryptophan and inhibit the production of kynurenine, thereby reducing the host’s inflammatory response levels (Gonçalves et al., 2022; Devereaux et al., 2024). These findings suggest that HSYA ameliorates gut microbiota dysbiosis in NAFLD and indicate that its therapeutic effects may be mediated, at least in part, through modulation of the gut microbiota.

Further serum non-targeted metabolomics analysis revealed that HSYA modulates the metabolism of phenylalanine and the biosynthetic pathways of phenylalanine, tyrosine and tryptophan in NAFLD mice. Phenylalanine, an aromatic amino acid with physiological activity, is converted into tyrosine in the body. Both phenylalanine and tryptophan are essential amino acids that must be obtained through dietary intake. A study involving over 2,000 subjects reported significantly higher levels of phenylalanine, tyrosine and other amino acids in NAFLD patients compared to healthy individuals (Hasegawa et al., 2020). These findings suggest that the biosynthetic metabolism of phenylalanine, tyrosine and tryptophan plays a critical role in the pathogenesis of NAFLD, with their metabolic pathways closely linked to lipid metabolism in the liver (Chen and Vitetta, 2020; Masoodi et al., 2021; Teunis et al., 2022). In this study, HSYA treatment significantly reduced tyrosine levels in NAFLD mice, indicating its ability to regulate metabolic disturbances involving tyrosine and related amino acids. This regulatory effect may represent a potential mechanism by which HSYA exerts its therapeutic effects on NAFLD. However, since this study did not include faecal microbiota transplantation experiments, we cannot conclusively confirm that HSYA improves serum metabolomic disturbances in NAFLD mice via modulation of gut microbiota. Future research will incorporate faecal microbiota transplantation to provide a more comprehensive understanding of the relationship between HSYA, gut microbiota and metabolomic regulation.

Pearson correlation analysis revealed a significant negative correlation between DL-tryptophan levels and the relative abundance of *Turicibacter*. Previous studies have demonstrated that *Turicibacter* in the mammalian gut reduces serum cholesterol, triglycerides and adipose tissue weight (Lynch et al., 2023; Yin et al., 2023; Zhao et al., 2023). In our study, HSYA treatment significantly increased the abundance of *Turicibacter* in the intestines of NAFLD mice, which was accompanied by significant improvements in oxidative stress and blood lipid profiles. Additionally, we observed a significant increase in serum phosphatidylcholine levels in the HSYA-treated group. Phosphatidylcholine plays a key role in phospholipid synthesis, promotes fat metabolism and inhibits hepatic fat deposition, thereby protecting the liver (Maev et al., 2020; Li et al., 2022c; Lu et al., 2022). These findings are consistent with previous research, further supporting the beneficial effects of HSYA on lipid metabolism and liver health in NAFLD mice.

This study revealed a significant positive correlation between serum N-formylmethionine levels and the abundance of *Erysipelotrichaceae* in the intestine. NAFLD mice exhibited elevated serum N-formylmethionine levels, which were significantly reduced following HSYA treatment. Previous research by Sigurdsson et al. (2022) reported that increased N-formylmethionine is often associated with activation of the pentose phosphate pathway and suppression of mitochondrial fatty acid β-oxidation. *Erysipelotrichaceae* are known to proliferate in the intestines of hosts experiencing high levels of inflammation (Chen and Vitetta, 2020), during which pro-inflammatory cytokine IL-1β expression and oxidative stress levels are significantly elevated (Bennett et al., 2020; Li et al., 2020). In this study, HSYA treatment significantly reduced the abundance of *Erysipelotrichaceae* in the intestines of NAFLD mice, coinciding with a decrease in serum N-formylmethionine levels. These findings suggest that *Erysipelotrichaceae* may play a pivotal role in the inflammatory processes of NAFLD and that HSYA mitigates inflammation by decreasing the abundance of this microorganism. The reduction in N-formylmethionine levels further indicates a decrease in oxidative stress and inflammation. However, the precise mechanisms by which *Erysipelotrichaceae* contribute to NAFLD pathogenesis remain to be elucidated through further investigation.

*Turicibacter* in the gut can also affect host glycerophospholipid metabolism and tryptophan/tyrosine synthesis metabolism (Chen et al., 2024). The results of this study suggest that the abundance of *Turicibacter* in the gut is positively correlated with the concentration of phosphatidylcholine in the serum and negatively correlated with the concentration of tyrosine. *Turicibacter* can express specific bile salt hydrolases (BSH) that participate in bile acid synthesis and metabolism, thereby affecting the host’s absorption and metabolism of lipids, including the metabolism of cholesterol, triglycerides, and phospholipids (Lynch et al., 2023). The study results indicate that the proliferation of *Turicibacter* may enhance lipid metabolic activity by promoting bile acid dissociation. Changes in the abundance of *Turicibacter* may affect tyrosine-related metabolic pathways through the “microbe-host co-metabolism axis”. The potential mechanism might be that the massive proliferation of *Turicibacter* inhibits the host’s tryptophan metabolism (kynurenine pathway) or phenylalanine metabolism, leading to a reduction in tyrosine synthesis precursors, thereby lowering the serum tyrosine concentration.

In conclusion, HSYA is a potent antioxidant capable of mitigating oxidative stress and inflammatory damage in the liver by modulating the gut microbiota and regulating metabolic pathways, including those of phenylalanine, tyrosine and tryptophan. These effects collectively contribute to the therapeutic potential of HSYA in treating NAFLD.

## Data Availability

The original contributions presented in the study are included in the article/Supplementary Material, further inquiries can be directed to the corresponding authors.
