# Supplementary material for: Hydroxysafflor yellow A alleviates oxidative stress and inflammatory damage in the livers of mice with nonalcoholic fatty liver disease and modulates gut microbiota
Source: Front Pharmacol. 2025 Jun 6;16:1568608. doi: 10.3389/fphar.2025.1568608 (PMC12179081; doi:10.3389/fphar.2025.1568608)
Supplement: Supplementary file 1 [file Supplementaryfile1.docx]

Supplementary Material

**S1 Construction of a NAFLD model in mice and administration of HSYA**

6-week-old SPF-grade male ICR mice (25g±4g) were provided by Jiangsu Wukong Biotechnology Co., Ltd. (Nanjing, China) and housed at the Experimental Animal Center of Jiangsu University. The feeding environment temperature was maintained at 20℃ to 26℃, with a relative humidity of 50% to 60%. Forty mice were randomly divided into 4 groups, with 10 mice in each group, including the normal control group (NC group), non-alcoholic fatty liver disease group (NAFLD group), high-dose hydroxysafflor yellow A intervention group (HSYAH group), and low-dose hydroxysafflor yellow A intervention group (HSYAL group). Mice in the NC group were fed a regular diet, while mice in the NAFLD group, HSYAH group, and HSYAL group were induced with high-fat diet (Future Biotech, Beijing, China, Cat. No.: D12109C) to develop NAFLD. The high-fat diet contained 40 kcal% Fat, 11.25 gm Cholesterol, and 4.5 gm Sodium cholate. Mice were provided with sterile purified water ad libitum. The average body weight (g) changes of mice in each group were monitored during the experiment. Hydroxysafflor yellow A was purchased from Shanghai Duma Biotechnology Co., Ltd. (molecular weight 801.01, purity 98%). Mice in the HSYAL group and HSYAH group were orally gavaged daily with doses of 60 mg/kg and 120 mg/kg, respectively, while the other two groups were given equal volumes of saline solution daily by gavage until the end of the 12th week of the experiment. After finishing the experimental periods’ mice were euthanized by intraperitoneal injection of Urethan (Sigma-Aldrich, St. Louis, MO, USA, 700 mg/kg). Mouse serum, liver, and colon contents are stored at -80°C, while the liver is fixed in 4% paraformaldehyde for use in paraffin sections.

**S2 Detection of indicators in mouse serum and liver**

100 mg of liver tissue from each group of mice were grind with a glass homogenizer. The supernatant was used for the detection of superoxide dismutase (SOD) and malondialdehyde (MDA). The SOD activity assay kit and the content MDA assay kit were provided by Jianjing Institute of BioEngineering (Nanjing, China). The testing procedure was carried out according to the kit operating instructions. Each group of mice serum was used for the detection of ALT, AST, TG, and TC concentrations.

**S3 Detection of inflammatory factor-related gene expression levels in mouse liver**

Using qRT-PCR to detect the expression levels of inflammatory factors mRNA in the liver. The liver of mice were quickly ground in a pre-cooled glass homogenizer to extract total RNA, which was then reverse transcribed into cDNA. The total volume of qRT-PCR was 20 μL, with 10 μL of SYBR Green Master pre-mix (Vazyme, Nanjing, China), 0.4 μL of upstream and downstream primers (10 μmol/L), and 2 μL of cDNA template. The reaction program included an initial denaturation at 95°C for 5 minutes, denaturation at 95°C for 3 seconds, annealing at 58°C for 20 seconds, extension at 72°C for 30 seconds, for a total of 40 reaction cycles. Using the mouse GAPDH gene as an internal reference, the relative expression levels of the target gene mRNA were calculated using the 2^−∆∆Ct^ formula. The sequence of qRT-PCR primers was shown in Table 1.

Table 1. qRT-PCR primers sequences.

| Genes | Primer sequences（5'→3'） |
| --- | --- |
| *Mouse_GAPDH* | F: CATCACTGCCACCCAGAAGACTG  R: ATGCCAGTGAGCTTCCCGTTCAG |
| *Mouse_IL-1β* | F: CCTGTCCTGCGTGTTGAAAGA  R: GGGAACTGGGCAGACTCAAA |
| *Mouse_NLRP3* | F: AACAGCCACCTCACTTCCAG  R: CCAACCACAATCTCCGAATG |
| *Mouse_Caspase-1* | F: GCACAAGACCTCTGACAGCA  R: TTGGGCAGTTCTTGGTATTC |
